# Digital referral experiences within Australian general practice: Insights from Chronic Obstructive Pulmonary Disease (COPD) care as an exemplar

**DOI:** 10.1371/journal.pone.0344663

**Published:** 2026-04-10

**Authors:** Thenuja Jayasinghe, Hancy Issac, Linda Deravin, Daniel Terry

**Affiliations:** 1 School of Nursing and Midwifery, University of Southern Queensland, Toowoomba, Queensland, Australia; 2 Southern Cross University, East Lismore, Queensland, Australia; 3 Gold Coast University Hospital, Southport, Queensland, Australia; 4 Institute for Health Research, University of Southern Queensland, Toowoomba, Queensland, Australia; 5 Institute of Health and Wellbeing, Federation University Australia, Ballarat, Victoria, Australia; Universiti Sains Malaysia, MALAYSIA

## Abstract

**Background:**

Digital referral platforms can strengthen communication between primary and specialist care and improve continuity for people with COPD. However, adoption in Australian primary care remains uneven across jurisdictions.

**Objective:**

To explore general practitioners’ (GPs) and practice managers’ (PMs) perspectives on barriers and enablers to adopting digital referral systems for COPD management in Australia.

**Materials and methods:**

A qualitative study was conducted with 16 participants (ten general practitioners and six practice managers) from urban, regional, and rural settings across five Australian states and territories. Semi-structured interviews (30–45 minutes) were conducted via Microsoft Teams and analysed using Braun and Clarke’s reflexive thematic approach. Inductive coding attended to role and location, and two researchers independently reviewed coding and interpretations to enhance confirmability. The COPD used as an exemplar to examine the barriers, enablers and system gaps associated with digital referral systems.

**Results:**

Four themes described current practice and needs. First, disrupted digital workflows: recurrent technical issues, limited interoperability with existing systems, and gaps in training reduced routine use. Second, fragmented communication: referrals often moved in one direction, with poor visibility of status and minimal feedback to primary care. Third, pragmatic enablers: auto-filled templates, transparent triage processes, and waiting time tracking reduced workload and uncertainty. Fourth, aspirations for integration: participants prioritised cross-sector interoperability, inclusive co-design, and real-time two-way messaging to support continuity, accountability, and timely care.

**Conclusion:**

Study participants described Australia’s digital referral landscape as fragmented, inconsistently adopted, and hindered by weak feedback loops. Usability features that automate data entry and surface referral status show immediate value and may accelerate uptake. Realising system-level benefits will require nationally coordinated policy, minimum interoperability standards, and targeted investment in regional, rural, and under-resourced settings. These practice-informed priorities translate front-line experience into actionable design and policy levers, offering a roadmap for procurement, co-design, and evaluation of digital referral platforms for COPD and other chronic conditions.

## Introduction

Effective referral pathways are essential in chronic disease management, supporting continuity of care and improving health outcomes. Chronic Obstructive Pulmonary Disease (COPD) is a progressive, non-reversible lung condition [1]. COPD was selected as an exemplar condition due to its complex care pathways, reliance on multidisciplinary collaboration, and frequent referrals between primary, secondary, and allied health services. By focusing on COPD, this study provides a lens through which to examine broader digital referral practices in Australian general practice, highlighting barriers, enablers, and systemic gaps that may apply across other chronic conditions.

In 2022, an estimated 638,000 Australians (2.5% of the population) were living with COPD, which ranks as the fourth leading cause of death globally [2,3]. In Australia, COPD accounted for 3.6% of the total disease burden and 50% of the respiratory disease burden, cost the health system $831.6 million in 2020–21, and caused 7,691 deaths (29.6 per 100,000) in 2022, representing 4% of all deaths.

A recent Australian GP survey reported that only 47% were familiar with the COPD-X Plan, and just half conducted patient reviews within seven days of discharge, with delays often linked to limited awareness of the admission. Additionally, 50% indicated that hospital discharge summaries lacked key clinical details. While most routinely assessed smoking status, immunisation, and medications, referrals to pulmonary rehabilitation and assessments of spirometry or oxygen therapy were less frequently prioritised [4]. These patterns align with wider evidence showing that acute exacerbations of COPD often result in emergency department presentations, with most patients discharged without admission, approximately 69% in the United States and 54% in Australia [5]. For those admitted, the post-discharge period is high risk, with 25% readmitted and 7% dying within 28 days. These outcomes are linked to limited self-management support, restricted GP access, and low pulmonary rehabilitation referral rates, with only 32% of eligible patients receiving a referral despite 77% having follow-up arranged [6]. Collectively, these findings highlight the need for integrated post-discharge pathways that connect tertiary and primary care, ensuring timely rehabilitation, proactive GP involvement, and targeted self-management support to reduce preventable readmissions and mortality.

In Australia’s universal, government-funded Medicare system, GPs act as gatekeepers to specialist and tertiary care. Access to publicly subsidised services requires a formal GP referral to ensure that care pathways are clinically justified, efficient, and aligned with broader health system priorities [7,8]. Practice Managers (PMs) support this process by overseeing clinic operations, managing referral workflows, and coordinating communication with external providers [9]. Effective collaboration between GPs and PMs through appropriate and timely referrals is critical for managing complex conditions such as COPD, which require coordinated input from specialists, allied health professionals, and community-based services like pulmonary rehabilitation.

However, many practices, particularly in regional and rural areas, still depend on outdated, one-way referral methods such as fax or scanned documents. These approaches lack feedback mechanisms, contributing to fragmented care and patient dissatisfaction [5,10]. Modern, integrated digital referral platforms have the potential to improve efficiency, communication, and continuity of care. Yet, without adequate interoperability, infrastructure, and user training, premature implementation risks disrupting workflows and worsening inequities in service access.

To address inefficiencies in traditional processes, several Australian jurisdictions have introduced digital referral platforms such *as Smart Referrals, HealthLink, Medical Objects, e-Referrals, Communicare, and electronic blue slips*. Often integrated with electronic medical records (EMRs), these systems aim to improve efficiency, transparency, security, and tracking [11,12]. However, uptake remains inconsistent due to system disintegration, poor interoperability, limited incorporation with hospitals and community services, clinician resistance, low digital literacy, and inadequate awareness or training [13]. Infrastructure limitations in regional and rural areas further exacerbate these challenges [14].

Many GPs continue to rely on manual referrals, not by choice but because of persistent technical and organisational barriers including siloed communication systems, unreliable platforms, inadequate feedback loops, and infrastructure gaps, particularly in rural and First Nations communities [15,16]. These issues reflect broader systemic, technical, and human-centred barriers, such as disjointed infrastructure, workflow disruptions, and limited stakeholder engagement [17]. Most existing digital platforms are unidirectional, requiring manual follow-up to confirm receipt and outcomes, which delays care [18].

Two-way digital referral systems, which enable seamless communication and feedback between GPs and specialists, have been shown internationally to improve efficiency, reduce fragmentation, and enhance patient outcomes [19–21]. Although Australian clinicians acknowledge their potential, particularly for chronic disease management such as COPD, adoption remains limited. Unresolved interoperability, infrastructure, and training challenges raise concerns that premature implementation may disrupt workflows and widen inequities [17,22].

Critically, little is known about how referral processes are experienced by GPs and PMs, which constrains the development of scalable, interoperable, and context-sensitive systems. This study addresses this gap by exploring COPD referrals in Australian general practice, identifying barriers and enablers of current digital systems, and generating insights to inform the co-design of practical, patient-centred solutions.

## Materials and methods

### Study design

This study adopted a qualitative interpretive approach to deeply explore how referral practices are experienced and understood within Australian primary care. Guided by a constructionist ontology and an interpretivist epistemology, it acknowledges that reality is co-constructed through social interaction and that knowledge is inherently shaped by context and lived experience [23]. This philosophical foundation supported the use of qualitative methods to examine the complex social and organisational processes influencing the implementation of digital referral systems. The study adhered to the Consolidated Criteria for Reporting Qualitative Research (COREQ) guidelines (S1 File)

**Fig 1:**
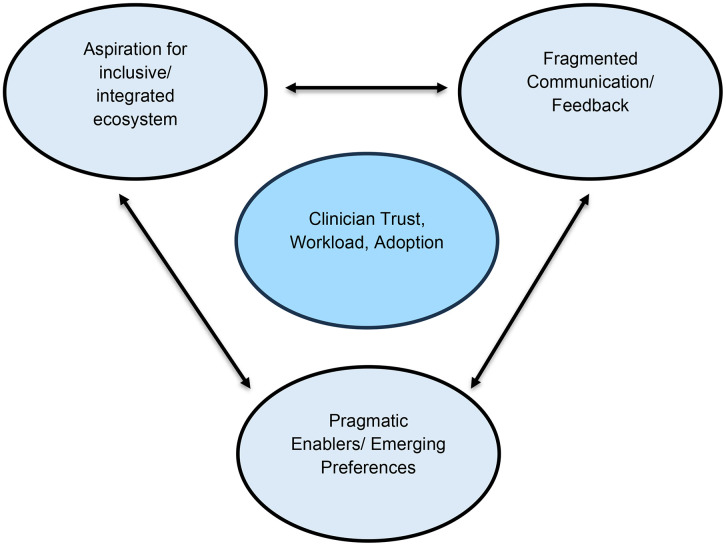
Thematic map illustrating interconnections among key themes.

### Setting and context

The study was conducted in general practice settings across both urban and rural areas in five of Australia’s eight states and territories, providing a broad representation of healthcare contexts and service delivery environments. These settings encompassed diverse practice sizes, patient populations, and resource availability, offering a varied backdrop for exploring experiences with COPD care and digital referral systems.

### Participants

Participants were general practitioners (GPs) and practice managers (PMs) who were currently involved in COPD care and had experience using digital referral systems. Inclusion criteria required active engagement in clinical or administrative aspects of COPD management within general practice. Individuals who were not currently practicing or who lacked familiarity with digital referrals were excluded to maintain the relevance and quality of the data [24]. A total of (16) participants were recruited, comprising (10 GPs and 6 PMs), with representation from both metropolitan, regional and rural practices.

While traditional approaches often rely on data saturation to determine sample adequacy, this study followed Braun and Clarke [25] Reflexive Thematic Analysis (RTA) which rejects the notion of data saturation as a maker of completion and adopts meaning-making as an ongoing, interpretive process rather than a definite endpoint. In keeping with Braun and Clarke’s RTA, this study conceptualised sample adequacy through information power by Malterud and colleagues [26]. Recruitment was guided by both the diversity and depth of insights required to answer the research question. However, practical and contextual constraints, including limited response rates despite multiple follow-ups, also influenced the final sample size.

### Recruitment

Recruitment was initiated through purposive snowball sampling through professional networks and extended via targeted outreach using email and social media platforms such as LinkedIn and Facebook. This mixed recruitment strategy was selected to ensure broad reach and engagement with diverse participants, particularly those with direct experience in digital referral processes [27]. Of the 35 invitations distributed, 16 participants consented, 6 declined, and 13 did not respond. To ensure a diverse participant pool while upholding ethical standards, the recruitment process included tailored invitations and measures to safeguard participants’ privacy and confidentiality. While snowballing enabled access to information-rich participants, it may have introduced homogeneity within professional circles. This limitation was mitigated through reflexive consideration of sample diversity, and adherence to qualitative trustworthiness principles of credibility, transferability, dependability, and confirmability. None of the participants were known to any of the authors.

### Data collection

Semi-structured interviews were conducted one-on-one by the lead author (TJ), a female Registered Nurse with five years of experience in general practice, as part of her higher degree by research training. Interviews were conducted between 1^st^ August 2024–5^th^ September 2024. Each interview lasted between 30 and 60 minutes and were video/audio recorded via Microsoft Teams. This platform was selected for its transcription capabilities and its ability to connect geographically dispersed participants. Interview discussions centered on digital transformation, interoperability, and the integration of clinical workflows in referral processes [28]. The flexible format allowed participants to reflect on their real-world experiences, revealing perceived challenges, benefits, and contextual influences associated with digital referrals in COPD care [29]. Field notes were also taken to support transcription accuracy and enrich contextual interpretation. Participants were offered the opportunity to review their interview transcripts; however, none elected to do so.

### Interview guide

Interview questions were developed based on a review of current literature and further refined through input from subject matter experts and feedback from the supervisory team to ensure clarity, relevance, and alignment with the objectives of the study. The questions were piloted with 10 GPs and 6 PMs and iteratively adjusted to enhance their relevance, clarity, and ethical appropriateness [30].

### Researcher reflexivity

The research team comprised experienced nursing and public health academics with backgrounds in rural, reginal and metropolitan health, chronic disease management, and qualitative research. Awareness of team’s professional perspectives informed the design and interpretation of the study. To minimise bias, the study used a semi-structured interview guide, engaged in regular peer debriefing, and maintained an audit trail throughout the analysis process. Reflexivity was conceptualised not as bias minimisation but as an interpretive practice, acknowledging that the researchers’ professional perspectives inevitably shaped analytic engagement. Regular discussions fostered critical awareness and deepened interpretation, consistent with Braun and Clarke’s [31] reflexive thematic analysis framework.

### Data management

All interview recordings and transcripts were stored on a secure, password-protected university server. Participant identifiers were removed during transcription, and each transcript was assigned a unique code (e.g.,-’GP4, NSW’ or ‘PM2, VIC’). Only authorised research team members had access to the data, which were managed in accordance with institutional ethics requirements.

### Data analysis

Reflexive thematic analysis was used to analyse interview transcripts. The six-phase approach involved familiarisation with the data, initial coding, theme development, reviewing and refining themes, defining themes, and producing a final report [25]. All transcripts were verified for accuracy and assigned unique identifiers based on participant role, interview sequence, and state location. For example, identifiers such as ‘GP4, NSW’ or ‘PM2, VIC’ were used to provide contextual depth and support interpretation of participant quotes.

Transcripts were manually coded using Microsoft Excel (Version 2507), allowing for flexible and immersive engagement with the data. Codes were generated inductively and then grouped into broader themes that reflected patterns across participants’ experiences. Coding was initially performed by the lead researcher and subsequently reviewed in collaboration with other researchers involved in the study to ensure rigour, consistency, and credibility [32]. Themes were iteratively refined to ensure coherence, analytical depth, and alignment with research objectives. Quotes were selected to illustrate each theme, enhancing credibility and confirmability. Regular supervisory meetings and reflective discussions supported the rigour and dependability of the analysis.

### Study rigour

Credibility was supported through peer debriefing within the research team and ongoing reflection on emerging findings. Dependability was maintained by following a consistent data collection protocol and documenting all methodological decisions in an audit trail. Confirmability was enhanced by retaining de-identified transcripts and analytic memos for review, ensuring interpretations were grounded in participant data. While transferability was supported through detailed descriptions of the study context, participant characteristics, and recruitment process, the inherently interpretive nature of RTA [25] limits the extent to which findings can be applied beyond this specific context. This has been acknowledged as a study limitation.

### Ethical considerations

Ethical approval for the study was granted on 29^th^ September 2023 by the University of Southern Queensland Human Research Ethics Committee (HREC Approval Number: ETH2023−0488). All participants received a detailed participant information sheet outlining the study’s purpose, procedures, risks, and benefits. Written informed consent was obtained prior to participation, and verbal confirmation was reconfirmed immediately before each interview, in accordance with institutional guidelines and the Declaration of Helsinki. Participants were given adequate time to consider their involvement and were encouraged to ask questions. They explicitly agreed to audio and/or video recording and to the use of de-identified quotes in publications. Participants were reminded of their right to withdraw at any time without consequence. All data were stored securely on password-protected servers accessible only to the research team and will be retained for five years in accordance with institutional policy. No vulnerable populations were involved. The study adhered to ethical reporting standards for qualitative research, including transparency in consent documentation and data handling.

## Results

Participants discussed digital referral processes in the context of COPD management across diverse clinical and geographical settings, including urban, regional, and rural practices. While the findings are based on COPD-related experiences, many of the identified barriers and enablers, such as interoperability challenges, feedback limitations, and administrative burden reflect broader issues in digital referral systems. Using COPD as an exemplar condition, the results illustrate both disease-specific nuances and general system-level constraints influencing referral effectiveness.

A total of 16 participants comprised of ten GPs (63%) and six PMs (37%) from Queensland (QLD), New South Wales (NSW), Victoria (Vic), Western Australia (WA), and the Australian Capital Territory (ACT). More than half were based in metropolitan areas (n = 10, 63%), with others in regional (n = 4, 25%) and rural settings (n = 2, 12%). GPs reported varied use of digital platforms such as *Smart Referrals, HealthLink, and Medical Objects,* depending on referral type and practice infrastructure. Nearly half (n = 7, 44%) worked in high-volume or specialised general practice settings, such as outreach clinics and aged care services, that cater for patients with chronic illnesses, socio-economic challenges, or experiences of homelessness. Most PMs (n = 5, 83%) managed dual systems involving both fax and digital tools due to software incompatibilities and provider preferences.

In addition, there was a varied level of experience with digital referrals, including preferences for system flexibility, concerns about interoperability, and challenges with outdated or unreliable technologies. COPD-specific insights reveal differing levels of engagement with referral platforms, with some participants noting improved triage and visibility, while others reported issues such as attachment rejections, lack of feedback, and system rigidity. These findings highlight the complexity and variability of digital referral practices in COPD care across Australian general practice settings (Table 1).

**Table 1:** Participant characteristics and summary of findings.

Participant	Setting	State	Digital Platforms Used	Key Focus / Comments	COPD Focus
GP1	Rural	QLD	Best Practice, Smart Referrals, Medical Objects, Fax	• Prefers narrative-based over tick-box systems • Wants interstate and allied health integration	• Smart Referrals for COPD but stresses system flexibility and timely attachments
GP2	Metropolitan	NSW/ ACT	Best Practice, HealthLink, HealthShare (lookup), Fax, Email	• Uses HealthLink real-time • Critiques fax as outdated and insecure	• Uses HealthLink for COPD • Challenge in tracing shared data across boarders
GP3	Rural	QLD	Best Practice, Smart Referrals	• Works with vulnerable patients • Critiques one-way systems • Seeks empathetic, feedback-rich design	• Uses Smart Referrals for COPD • Highlights complexity in comorbidity referrals
GP4	Rural	NSW	Best Practice, HealthLink, Fax	• Wants cross-state interoperability • Supports automation and voice-to-text	• HealthLink for COPD but lacks tracking • Refers across borders
GP5	Metro	QLD	Best Practice, Smart Referrals, Medical Objects, Fax	• Supports Smart Referrals with triage and wait time features • Highlights limitations with file sizes	• Uses Smart Referrals for COPD • Notes improved visibility and triage
GP6	Regional	NSW	Best Practice, SENT, HealthShare (past), Fax, Email	• Prefers SENT triage, Requests interoperability • Flags rigid workflow criteria	• Refers COPD patients via SENT • Notes limited catchment communication
GP7	Metropolitan	Vic	Best Practice, HealthLink, Argus, Fax, Email	• Transitioning to digital • Feedback and upload issues	• Refers COPD to public clinics via HealthLink but still uses fax
GP8	Regional	QLD	Best Practice, Smart Referrals, Medical Objects, Fax	• Favors Medical Objects for document retention • Finds Smart Referrals slow, clunky, glitchy	• Uses Smart Referrals for COPD if meets criteria; uses Medical Objects when rejected
GP9	Remote	WA	WACHS eReferral, Communicare, Email, Fax	• Notes no linkage with EMRs • Manual, siloed data entry	• Refers COPD via WACHS eReferrals • Notes high burden of comorbidities
GP10	Metro	QLD/ ACT	Best Practice, Smart Referrals, Medical Objects, Fax (rare)	• Mixed views on Smart Referrals • Requests override and better workflow features	• Supports Smart Referrals for COPD, but flags poor integration with private diagnostics
PM1	Rural	NSW	Medical Director, HealthLink, MD Exchange, Fax, Email	• Uses digital fax via email • Highlights fragmentation and loss of letters	• Coordinates COPD care with GPs • Sees gaps in feedback from hospital
PM2	Regional	QLD	Best Practice, Medical Objects, Smart Referrals, Email, Fax	• GPs use Smart Referrals, admins don’t • Wants acknowledgment and tracking systems	• Tracks COPD referrals via GP inboxes • Notes training inconsistency
PM3	Regional	QLD	Best Practice, Smart Referrals, Medical Objects, Fax	• Refers for allied health using GPMPs • Smart Referrals slow, crashing often	• Refers COPD to public via Smart Referrals • Fax still common
PM4	Regional	QLD	Medical Objects, Argus (past), Email, Fax	• Strongly favors Medical Objects • Dislikes fax for privacy • Audits undelivered letters	• Follows up COPD patients referred from external GP clinics to specialist clinics
PM5	Regional	QLD	Best Practice, Smart Referrals	• Trains GPs • Flags upload issues and software delays	• Sees COPD as one of many referral areas • Issues with attachment rejection common
PM6	Regional	QLD	Best Practice, Smart Referrals, Fax	• Describes Smart Referrals as clunky • 50% GP uptake • Reverts to fax when stuck	• Manages hundreds of COPD patients • Referrals depend on patient consent and system flexibility

Participants discussed the implementation and use of digital referral systems in COPD management across diverse geographic and clinical contexts, including urban, regional, and rural general practices. Their experiences reflected varying levels of familiarity with digital platforms and infrastructure. Despite this diversity, several consistent challenges emerged. These included onboarding gaps, limited system integration, continued reliance on fax, and administrative burdens such as undeliverable referrals, outdated provider directories, and the absence of bidirectional communication. A thematic synthesis of these findings, illustrating key barriers and enablers through participant quotes (Table 2). The themes highlight critical issues such as interoperability limitations, inconsistent feedback mechanisms, usability concerns, and the need for more flexible, context-sensitive referral systems to support effective COPD care (Table 2).

**Table 2:** Thematic Findings Summary.

Major Theme	Sub-Theme / Finding	Summary of Insights	Supporting Quotes
1) Systemic Disruptions in Digital Workflows	Digital Referral Systems	Widespread use of Smart Referrals, HealthLink, Medical Objects and varied platforms by setting and infrastructure, though system differences affect reliability.	“Smart Referrals is mandatory for public patients...” (PM5, QLD) / “I use HealthLink while the patient is with me...” (GP2, NSW/ACT) / “Smart Referrals often doesn’t save the referral letter...” (GP8, QLD) / “The platforms don’t talk to each other; I re‑enter data.” (GP9, WA) / “Outages early on put me off using it.” (GP5, QLD)
	Fax vs Digital	Fax remains in use due to habits, provider limitations, and tech failures despite digital availability.	“We still use fax due to provider turnover...” (PM3, QLD) / “Fax is still seen as safe but outdated...” (GP1, QLD) / “Some specialists don’t provide emails, so we fax.” (GP10, QLD) / “Hospitals still prefer fax for some referrals.” (GP8, QLD) / “I don’t like fax, but at least it’s received.” (GP9, WA) / “I can’t guarantee fax goes to the right destination.” (PM5, QLD)
	Barriers to Implementation	Common issues include upload limits, unstable interfaces, and redundant manual entry requirements.	“File size limits discourage uploads...” (PM5, QLD) / “Smart Referrals doesn’t save letters...” (GP8, QLD) / “If Smart Referrals is down, we rely on Medical Objects... it adds steps.” (GP10, QLD) / “Hospitals can’t read investigations, so we fax them again.” (GP8, QLD) / “Delivery reports can be delayed 24–48 hours.” (PM2, QLD)
	Integration Challenges	Digital systems often lack full integration, creating inefficiencies and duplicate workflows.	“Smart Referrals clashes with our system...” (PM6, QLD) / “Some specialists - ren’t even in the system...” (GP6, NSW) / “Smart Referrals needs better integration with Best Practice.” (GP1, QLD) / “Lack of interoperability makes the process cumbersome.” (GP9, WA) / “Not all specialists are listed on HealthLink.” (GP6, NSW)
2) Fragmentation of Communication and Feedback	Continuity of Care	Delayed or absent feedback from specialists and hospitals undermines follow-up and patient outcomes.	“Feedback is helpful but not always timely...” (GP4, NSW/ACT) / “Patients return saying they heard nothing back.” (GP7, VIC) / “We rarely get feedback from specialists.” (GP8, QLD) / “Patients haven’t heard from the hospital; we haven’t either.” (GP10, QLD) / “No real‑time communication; like a black hole.” (GP4, NSW/ACT)
	Policy and Feedback	GPs and PMs call for urgent tags, real-time updates, and transparent referral tracking.	“No urgent tag option in Medical Objects...” (PM4, QLD) / “Feedback isn’t consistent unless followed up.” (GP7, VIC) / “A live chat between GPs and specialists would help.” (GP5, QLD) / “A two‑way system would keep us informed.” (GP5,QLD) / “Policy changes should make referrals smoother, transparent.” (GP10, QLD)
3) Pragmatic Enablers and Emerging Preferences	Training and Usability	Initial training is inadequate; ongoing learning occurs on the job, affecting adoption consistency.	“Training is minimal - we learn as we go...” (GP9, WA) / “New GPs found it intimidating...” (PM5, QLD) / “Training is crucial; without it, staff struggle.” (PM5, QLD) / “Proper training would save time and improve efficiency.” (GP1, QLD) / “Training on the system would improve accuracy.” (GP10, QLD)
	Rational Thinking vs Tick-box	Rigid systems restrict nuanced cases; participants prefer a balance of automation and flexibility.	“The system stops if a tick box is missed...” (PM6, QLD) / “Red box prompts are helpful but frustrating...” (GP5, QLD) / “No override for urgent cases delays care.” (GP, QLD) / “We need an override button for non‑standard patients.” (PM6, QLD) / “When Smart Referrals gets clunky, doctors go back to fax.” (PM2, QLD)
4) Aspirations for an Inclusive and Integrated Ecosystem	Allied Health Referrals, Interstate Referrals & Private Specialist Referrals	COPD care relies on access to services like pulmonary rehab, often unavailable in rural settings.	“We refer via GPMPs and Medicare pathways...” (PM3, QLD) / “Pulmonary rehab access is limited...” (GP9, WA) / “Rehab is mostly metropolitan, not rural.” (GP9, WA) / “Rural patients need a respiratory physician to access rehab.” (GP2, ACT) / “Rural areas are underserved; visiting nurses are the best we have.” (GP9, WA) / “One reason per visit doesn’t fit complex cases.” (PM6, QLD) / “Homeless patients lack addresses; follow‑up letters miss them.” (GP3, QLD)

Within the context of these shared experiences, four overarching themes emerged that illustrated both the practical realities and aspirational possibilities of digital referral use in COPD care. These themes included (1) Systemic disruptions in digital workflows, (2) Fragmentation of communication and feedback, (3) Pragmatic enablers and emerging preferences, and (4) Aspirations for an inclusive and integrated digital referral ecosystem. Together, they reveal the practical challenges and transformative potential of digital referrals when aligned with user needs, system capacity and patient care priorities and are discussed in detail.

Fig 1 demonstrates how workflow disruptions, feedback fragmentation, and system usability challenges collectively affect clinicians’ trust, workload, and adoption of digital referral systems. It visually integrates the study’s three core themes, showing their overlapping influence on the overall experience of digital referral implementation in COPD care. Fig 1 further emphasises identified challenges are mutually reinforcing, highlighting the need for system-wide approaches rather than isolated technological fixes. Within this context each of the themes are discussed in detail.

### Systemic disruptions in digital workflows

Participants frequently described digital referral systems as unpredictable and difficult to integrate into daily workflows. While platforms such as Smart Referrals, HealthLink, Medical Objects, and Communicare offered structured formats and embedded logic, they often failed to perform reliably in real-world practice. System crashes, file upload restrictions, navigation issues, and misalignments between platform capabilities and clinical needs were commonly reported.

GPs and PMs described how digital referral tools, despite their potential, created operational inefficiencies. Participants felt these systems lacked interoperability, required significant manual workarounds, and offered limited flexibility during referral creation.

One GP noted, *“Smart Referrals can be hit or miss... it doesn’t always work smoothly, particularly when the hospital system is not fully integrated”* (GP1, QLD). Another participant reported that their platform required manual re-entry of patient details, stating, “*It doesn’t self-populate... it’s still a blank page*” (GP9, WA).

Training and onboarding also contributed to limited adoption. New users were often expected to self-navigate the systems or rely on informal support. This approach led to inconsistent use, especially in practices where there was high turnover of GPs, or those serving complex health populations. As one participant explained, *“Training is minimal; we learn as we go”* (GP9, WA), while another noted that *“Proper training would save time and improve efficiency”* (GP1, QLD). The lack of structured onboarding not only reduced confidence and accuracy but also amplified workload pressures, reinforcing perceptions that digital referral systems were inefficient and burdensome.

These recurring breakdowns extended beyond technical inconvenience; they disrupted professional routines, diminished clinicians’ sense of control, and eroded trust in the reliability of digital referral tools that were intended to streamline practice. Limited training and onboarding compounded these issues, as new users were often expected to self-navigate unfamiliar systems or rely on ad hoc peer support. The combination of technical fragility and inadequate preparation heightened frustration and reinforced perceptions that digital referrals increased workload rather than improved workflow efficiency.

### Fragmentation of communication and feedback

Participants consistently highlighted gaps in information flow that undermined continuity of care. A persistent theme across interviews was the unidirectional nature of digital referrals reflecting broader issues of fragmented communication between primary and secondary care. While referrals could be submitted digitally, feedback regarding their receipt, triage, or outcome was infrequent or absent. GPs and PMs described a communication void with one participant noting:

“*It feels like sending something into a black hole; you don’t know if it’s been seen, let alone what happens next*.” (GP4, NSW/ACT).

That placed additional burden on administrative staff and jeopardised follow-up care. This disintegration led to concerns about incomplete patient journeys and missed appointments. Respective participants expressed that they could not confirm whether referrals had been acted upon unless patients returned or manual follow-up was initiated. As one GP stated,

“*Once you refer the patient, it’s off your hands… you might not see the notes unless you follow up intentionally”* (GP9, WA). A PM added, *“Feedback isn’t consistent unless followed up”* (PM2, QLD).

Fax remained in use, largely because it offered perceived reliability and confirmation of receipt, particularly when digital platforms failed due to technical issues, poor training, or systemic outage despite its inefficiency and lack of data security. One participant noted,

*“Referrals were paper-based, printed and faxed - at least with fax you know they’ve received something*” (GP2, NSW/ACT). Others echoed similar sentiments: *“Technical issues or system outages affect eReferral reliability, so a backup method is always necessary*” (GP4, NSW), and *“Fax is still used out of habit and lack of training in digital platforms, despite being outdated and error-prone*” (GP7, VIC).

Participants also criticised the rigid structure of referral templates. Many systems required fields to be completed even when they were not clinically necessary. Inflexibility limited the ability to personalise referrals, particularly in urgent or nuanced cases. One PM explained:

“*It’s difficult to pin down one reason for a visit, patients often have multiple issues, but the system forces you to choose one, even if it’s not the main concern.”* (PM6, QLD).

The lack of feedback and communication transparency extended beyond administrative inconvenience; it shaped clinicians’ sense of accountability and trust in the referral process. Without confirmation of receipt or outcome, many participants described feeling “disconnected” from patient progress and uncertain about whether referrals were acted upon. This disconnection reinforced the perception of digital referrals as one-way transactions rather than collaborative tools for continuity of care. The cumulative effect of unreliable feedback mechanisms and poor system interoperability compounded clinicians’ workload and undermined confidence in the broader promise of digital health integration.

### Pragmatic enablers and emerging preferences

Despite the barriers, several features were identified as enablers of effective digital referral use. Automation, particularly the auto-population of patient information and test results, reduced administrative workload and enhanced accuracy.

“*Once you open the patient’s file, you click on HealthLink, and then the data is transferred. You don’t have to manually fill anything. It automatically fills your investigations*” (GP7, VIC). Similarly, another stated *“Even if you’re new to it, it’s user-friendly. I think it’s idiot-proof”, and another GP added “If Communicare could populate the eReferral platform, like history, medications, allergies, it would be smooth. That’s the beauty of integration*” (GP9, WA).

Participants praised the clinical logic embedded in some platforms, especially Smart Referrals. For example, the system could prompt users to include mandatory investigations such as spirometry, improving referral completeness. As one GP explained,

“*The essential referral information, particularly the investigations that are necessary, automatically get sucked out of the practice software… you can attach spirometry results easily with Smart Referrals”* (GP5, QLD).

Participants appreciated referral systems that offered suggestions based on patient proximity as well as visibility into triage categories and hospital wait times. These features supported clinical decision-making, streamlined referral choices, and enhanced communication with patients about expected timelines and service availability.

“*The ability to see referral wait times by category and hospital has improved GP decision-making*” (PM5, QLD). In addtion, another participant explained, “*I like the idea that you can see the category of the hospital and how long the wait time is. That actually helps us explain things to the patient better*” (GP6, NSW).

These features helped reduce referral rejection and improved transparency in service access. However, usability varied significantly across states, and lack of interoperability between systems remained a major concern.

While features such as automatic data entry and referral tracking improved efficiency, their benefits were undermined by ongoing interoperability issues and inconsistent usability across states. The combined effects of technical failures and poor feedback mechanisms continued to disintegrate clinicians’ confidence. Despite some progress, limited integration and uneven implementation highlighted the need for a nationally aligned, user-informed digital referral system.

### Aspirations for an inclusive and integrated ecosystem

Across the interviews, there was a clear aspiration for a nationally consistent and integrated referral ecosystem. Participants envisioned platforms that connected public and private sectors, offered two-way communication, and embedded feedback mechanisms that closed the referral loop.

Several GPs expressed frustration that their systems only covered public hospital pathways, forcing them to maintain parallel processes for private referrals. This often-involved manual duplication of information and reliance on fax or email for private sector communication. One GP explained, “*Smart Referrals is limited only to the public system, so you still have to write a separate referral letter for private*” (GP8, QLD).

Participants also described the need for referral platforms to better support patients from underserved populations, including First Nations communities and individuals experiencing homelessness. The inability to track referral outcomes or receive discharge summaries created considerable gaps in care continuity for these patients. For example, a GP working with homeless populations remarked, “We need a two-way system that actually gives us updates, especially for patients experiencing homelessness who don’t have stable addresses or phones, which complicates follow-up care” (GP3, QLD).

In addition, a PM added: *There needs to be better testing and implementation strategies, particularly for Aboriginal Medical Services, as Smart Referrals often fail to accommodate the complexity of our patients and do not always align with their needs. It is also difficult to capture accurate data about COPD during a single consult due to the multitude of issues typically addressed.* (PM6, QLD)

This theme also captured frustrations with cross-jurisdictional barriers. For example, GPs practicing in border towns between states described being unable to seamlessly refer patients to services across state lines. One GP explained

*The system works well for referrals within our state, but if we need to refer to a hospital in the neighbouring state, we’re back to printing and faxing. It feels like we have one foot in digital and the other in the 1990s.* (GP4, NSW)

While recognising that referral systems in Australia are state-based and do not currently interlink, participants across jurisdictions reported similar frustrations with fragmentation, unidirectional communication, and inefficiencies.

Overall, participants described a healthcare environment where digital tools offered promise but often required manual workarounds and supplemental processes to ensure safe and effective care. In addition, participants consistently advocated for platforms that are reliable, intuitive, and interoperable across states, sectors, and systems.

These usability constraints heightened cognitive and administrative workload, prompting clinicians to question whether digital systems genuinely supported rather than complicated their clinical autonomy and patient-centred decision-making.

The lack of system interoperability compounded administrative burden and diminished clinicians’ trust in digital referral tools. Collectively, these interconnected challenges generated a sense of technological fatigue and highlighted the urgent need for an integrated, context-sensitive referral ecosystem. This relationship among themes is depicted in Fig 1, which illustrates how technological fragility, communication gaps, and workflow pressures converge to shape clinicians’ engagement with digital referral systems in COPD care.

Although participants described common challenges across Australia, variations in digital referral policies and system infrastructure between states influenced their experiences. For example, clinicians in Queensland and Victoria reported differing levels of integration with My Health Record and local Primary Health Networks. These jurisdictional inconsistencies affected interoperability, feedback mechanisms, and the perceived usability of digital platforms.

Across all themes, participants envisioned an inclusive digital referral ecosystem linking primary, secondary, and allied health services through a single interoperable platform. Their aspirations reflected frustrations with disrupted workflows, fragmented feedback, and inconsistent usability across systems. Beyond policy reform, participants called for human-centred design, national interoperability standards, and equitable access across regions. These aspirations reflect a deeper professional yearning to restore trust, autonomy, and efficiency through genuinely integrated digital referral practices.

## Discussion

This study explored clinicians’ experiences with digital referral systems in Australian general practice, using COPD as an exemplar for understanding broader implementation challenges. The findings extend current knowledge by revealing how technological reliability, communication flow, and system usability interact to shape clinicians’ trust, autonomy, and engagement with digital tools. Rather than isolated technical or administrative issues, these interconnected factors reflect deeper tensions between clinical realities and digital design. Through a reflexive thematic lens, the study highlights that successful digital referral adoption depends not only on system functionality but also on how well these systems align with the values, workflows, and relational practices of healthcare professionals.

The experiences of GPs and PMs using digital referral systems for COPD management in Australia revealed key systemic challenges and emerging preferences in how referrals are managed across diverse clinical settings. The findings highlight that, while digital systems offer potential efficiency gains, their current implementation is hindered by technical fragmentation, policy inconsistency, and limited integration with real-world workflows [33].

Participants reported frequent system failures, limited upload capabilities, and poor integration between primary and secondary care systems. The continued reliance on fax machines reflects the persistence of these unresolved issues. Similar challenges have been reported internationally, including poor interoperability between EHR systems, slow system performance, limited device compatibility, and poorly designed user interfaces [34]. Organisational challenges, such as insufficient leadership, clinician resistance, and inadequate investment in training and infrastructure, also contribute to poor adoption [35].

These issues are further compounded recent evidence identifying inadequate training as a major contributor to low digital confidence, particularly among new users [36]. Smaller practices face greater challenges due to limited resources and disjointed workflows [37,38] where digital literacy gaps and unfamiliarity with system benefits further intensify clinician resistance to changing established work practices [35]. As a result, digital transitions can disrupt clinical workflows, with participants reporting increased task completion times during early adoption and cognitive strain from alert-heavy interfaces. These experiences align with concepts such as Digital Deceleration, Data Discordance, and Digital Hypervigilance, which describe user fatigue and inefficiency arising from poorly integrated systems [39].

The absence of two-way feedback between primary and secondary care was identified as a major barrier to continuity and coordination of care. These experiences echo findings by Pocock et al. (2025) who emphasised the importance of closing the communication loop for integrated care. Underlying this fragmentation are incompatible EHR systems, lack of standardisation, and entrenched institutional boundaries that inhibit seamless information flow [40,41].

These systemic deficiencies are particularly pronounced in rural contexts and First Nations communities, where disconnected communication systems exacerbate health inequities. This is especially problematic for COPD patients, who require coordinated, multidisciplinary care. Delays in feedback and reliance on informal channels such as fax and messaging apps introduce safety and quality risks [42]. Fax can result in misrouted or outdated documents, and often bypass governance mechanisms, leading to privacy breaches and untracked decision-making. In contrast, although digital referral platforms aim to streamline processes, poor design can create inefficiencies such as duplication, missing information or attachments, and overly rigid templates that disrupt workflow efficiency [43]. The persistence of informal workarounds highlights the need for socio‑technical design principles [44] and standardised referral protocols built in real-world workflows [19]. Broader system-level enablers, such as digital literacy, patient engagement, and system navigation support, are essential to promote equity and improve referral outcomes as opposed to merely expanding downstream access mechanisms [45,46].

Pragmatic enablers identified in this study, such as auto-fill functionalities, triage visibility, and integrated messaging, embody clinician-centred design that enhances usability and workflow efficiency. Recent implementations like the National Health Service in UK’s, automated triage features demonstrate how aligning referral systems with clinical criteria can reduce administrative delays [47]. International platforms like Scotland’s Scottish Care Information (SCI) Gateway and the Netherlands’ ZorgDomein demonstrate how incorporating these design elements can reduce missed appointments and enhance continuity of care [48]. However, participants in this study emphasised, achieving similar outcomes locally requires more than good design. Successful implementation depends on policy support, alignment with user needs, and infrastructure readiness to ensure systems are not only technically feasible but also practically adoptable across diverse contexts [49,50]. The importance of collaborative design is also recognised by Peters, Guccione [51] who highlight the value of frontline input, noting that co-design with healthcare professionals and consumers requires systematic evaluation to determine its overall impact. Effective onboarding, implementation of a digital referral algorithm and ongoing training further support digital transitions, particularly in high-need rural regions [17].

Participants envisioned an inclusive digital referral ecosystem that integrates public and private care, provides real-time feedback, enhances transparency, and equitably addresses individual needs. These goals align with global digital health principles that emphasise inclusivity, responsiveness, and user empowerment [52]. Achieving this vision depends on interoperability as a foundational element, enabling seamless data exchange across sectors and improving patient outcomes [53–55]. In addition, participants also advocated for private provider integration, override functions, and patient-access features. These ambitions reflect human-centred design principles [56,57] and highlight the need for policy harmonisation across states and sectors.

Australia’s current health system is characterised by public funding, mixed public–private provision, and large rural populations. These structural complexities limit the transferability of one- or two-way digital referral models. Federated governance further divides health responsibilities across states and territories, each operating its own digital infrastructure, referral protocols, and interoperability standards [16,58,59]. This decentralisation is compounded by incompatible EHR systems, inconsistent privacy and data governance regulations [60], and limited integration between public and private providers due to differing funding models and contractual arrangements [61]. Political and institutional inertia, together with intergovernmental negotiation delays, continue to hinder national reform [62,63]. Without a coordinated national strategy and enforceable digital standards, the feasibility of a single, scalable referral system in Australia remains restricted.

Addressing Australia’s digital divide requires targeted investment in equitable infrastructure, culturally responsive technologies, and sustained digital literacy initiatives particularly in rural areas where connectivity literacy is critical for meaningful digital inclusion [64]. Emerging innovations, such as Geographic Information Systems (GIS) and AI-powered analytics, offer promising potential to optimise referral pathways [65,66]. However, their successful deployment depends on robust governance structures (risk, data quality, bias, human oversight) and ethically informed implementation strategies [67–69].

The findings stress the need to co-design digital referral systems with end users, embedding them into existing clinical workflows, and support them through coherent policies, comprehensive training, and scalable infrastructure. Without these foundational elements, digital innovations risk reinforcing, rather than reducing, existing healthcare inequities particularly in chronic disease management and access to timely care. Healthcare will always centre on people – clinicians, patients, and carers – but its effectiveness increasingly depends on the systems that support them. For digital referrals to truly deliver, the infrastructure behind the scenes must be as responsive, intelligent, and adaptable as the people it is designed to serve. Achieving this requires not only technical innovation, but also thoughtful integration aligned with real-world clinical needs, workflows, and human relationships.

### Limitations

While the sample size was appropriate for qualitative inquiry, it may not fully capture the breadth of geographical and demographic diversity across Australia. Most participants were based in QLD and NSW, potentially limiting the representation of experiences from other states and territories. The use of purposive snowball sampling may have introduced selection bias, as individuals more engaged with digital referral systems or holding stronger views may have been more inclined to participate. This may result in underrepresentation of clinicians who are less digitally confident or disengaged from referral technologies. Although the study included participants from a range of general practice settings, rural, regional, and metropolitan, it did not incorporate perspectives from hospital-based clinicians, digital health platform developers, or respiratory specialists. While these groups were outside the scope of the focus of the study on general practice referral processes, their exclusion may limit the broader applicability of the findings across the wider referral ecosystem. As COPD was used as an exemplar condition, the findings should be interpreted as contextually situated insights into digital referral practices rather than disease-specific generalisations. This framing may limit direct transferability to other conditions but strengthens the conceptual understanding of system-level barriers and enablers in primary care digital referral implementation.

### Implications

The findings highlight the urgent need for coordinated national efforts to strengthen digital referral infrastructure, particularly for chronic disease management such as COPD. Key priorities include system interoperability, real-time feedback loops, and inclusive platform design that supports both public and private sector workflows. Addressing digital literacy and infrastructure gaps, especially in rural and underserved areas, will be critical to ensuring equitable access. However, achieving these improvements will require navigating the complexities of Australia’s state-based health systems, where digital infrastructure and policy maturity vary across jurisdictions. Collaborative engagement between federal and state governments, digital health developers, and clinical stakeholders will be essential to align priorities and ensure sustainable implementation. These insights can inform co-design initiatives, policymaking, and implementation strategies that reflect the lived experiences of frontline users and contribute to broader goals of health equity and system resilience.

## Conclusion

This study highlights the pressing need for coordinated and equity-focused reform to realise the potential of digital referral systems within Australian primary healthcare. While the findings provide valuable insights from general practitioners and practice managers, they reflect a small purposive sample and should therefore be interpreted within this participant context rather than as nationally representative. Despite this, digital tools offer opportunities to streamline chronic disease management, particularly for conditions such as COPD, and their success depends on more than technical functionality.

Achieving a seamless referral ecosystem requires system-wide interoperability, policy alignment, and meaningful engagement with frontline stakeholders. Importantly, digital referral systems must be designed to reflect the realities of clinical practice. This includes supporting the workflows, priorities, and lived experiences of GPs and PMs, who are central to their implementation. Inclusive design, real-time feedback mechanisms, and integration across public and private sectors are essential to ensure usability and uptake.

The findings also have highlighted the challenges posed by Australia’s state-based health system, where fragmented infrastructure and policy maturity vary across jurisdictions. Addressing these disparities, alongside digital literacy and infrastructure gaps in rural and underserved areas, is critical to ensuring equitable access and outcomes. Future research needs to explore cross-sector collaboration models, evaluate implementation strategies across different jurisdictions, and include perspectives from hospital-based clinicians, platform developers, and specialists to build a more comprehensive understanding of the referral ecosystem. By embedding co-design principles and aligning digital tools with clinical realities, Australia can move toward a more integrated, responsive, and equitable digital health system.

## Supporting information

S1 File COREQ Checklist. (PDF)
